# Clinical and Imaging Characteristics of Herlyn-Werner-Wunderlich Syndrome: a Comprehensive Analysis

**DOI:** 10.1007/s43032-024-01628-2

**Published:** 2024-06-21

**Authors:** Jiani Qi, Ping Zhou, Hong Peng, Jin Deng, Yang Shao, Lingjuan Ye, Shengjuan Luo

**Affiliations:** Department of Ultrasonography, The Third Xiangya Hospital of Central South University, Changsha, Hunan Province China

**Keywords:** Herlyn-Werner-Wunderlich syndrome (HWWS), Complete obstruction, Incomplete obstruction, Obstructed hemivagina, Congenital malformation

## Abstract

**Purpose:**

To describe the clinical and imaging characteristics of Herlyn-Werner-Wunderlich syndrome (HWWS).

**Methods:**

This study presented an observational case series involving consecutive patients diagnosed with HWWS, whose medical records were retrospectively reviewed. From June 2012 to December 2022, there were a total of 85 patients with HWWS enrolled in our study. We obtained the medical history, including demographic characteristics, clinical presentation, treatment, complications, and radiologic examinations performed. Patients > 18 years of age (*n* = 58) were recontacted.

**Result:**

In our analysis, 27 patients were categorised as having complete obstruction, and 58 were categorised as having incomplete obstruction. The mean age at the onset of symptoms and diagnosis of complete obstruction was significantly younger than incomplete obstruction (*P* < 0.05). For complete obstruction, the median time between menarche and the onset of symptoms was 2.1 years, while for incomplete obstruction, it was 5.3 years. There was a significantly lower incidence of intermittent mucopurulent discharge, irregular vaginal haemorrhage, and occasional examination findings of complete obstruction than incomplete obstruction (*P* < 0.05). Complete obstruction was significantly associated with dysmenorrhea and pelvic endometriosis compared with incomplete obstruction (*P* < 0.05).

**Conclusions:**

There are distinct clinical differences between patients with complete obstruction of the hemivagina and those with incomplete obstruction. HWWS can manifest as various combinations of uterine anomalies, communications anomalies, and renal anomalies. Early recognition and treatment can avoid complications and preserve fertility.

**Keyswords:**

Herlyn-Werner-Wunderlich syndrome (HWWS); complete obstruction; incomplete obstruction; obstructed hemivagina; congenital malformation.

**Supplementary Information:**

The online version contains supplementary material available at 10.1007/s43032-024-01628-2.

## Introduction

Herlyn-Werner-Wunderlich syndrome (HWWS), a rare female urogenital tract abnormality that was originally reported in 1922, is distinguished by uterine didelphys, blind hemivagina, and ipsilateral renal agenesis [1]. Herlyn, Werner, and Wunderlich gave a thorough description of this condition and gave it the acronym HWWS [2, 3]. OHVIRA (Obstructed Hemivagina and Ipsilateral Renal Anomaly) was postulated by Smith and Laufer in 2007 [4], which is equivalent to HWWS. In the female population, the incidence of HWWS varies from 0.1 to 3.8% or higher.

The intermediate mesoderm is where the genital and urinary systems first originated. The genital system develops in accordance with normal development of the Müllerian ducts. A wide variety of abnormalities, known as Müllerian ducts anomalies, can result from one of three processes, working alone or in combination: the stoppage of duct development, the failure of their fusion, or the inability to reabsorb the medial septum [5]. The top two-thirds of the female reproductive tract (the uterus, fallopian tubes, cervix, and upper vagina) are said to be caused by the Müllerian ducts (paramesonephric ducts), whereas the urinary system and lower vagina are, respectively, caused by the Wolffian ducts (mesonephric ducts) and urogenital sinus [6–8]. HWWS is caused by the disruption of embryology in the female urogenital tract. HWWS only exhibits a few generalized symptoms, such as varying degrees of dysmenorrhea, vaginal discharge, pelvic mass, or even acute abdomen [9]. Clinicians lack an in-depth understanding of HWWS due to its rarity and varied manifestations. Due to retrograde blood flow, several of these individuals had misdiagnoses and missed diagnoses, leading to certain major complications (endometriosis, haematosalpinx, pelvic inflammatory disease, etc.). The aim of this study is to increase knowledge and facilitate better detection of HWWS in the future by describing their clinical symptoms and imaging characteristics.

## Materials and Methods

This study presented an observational case series involving consecutive patients diagnosed with HWWS, whose medical records were retrospectively reviewed. The retrospective nature of the study was approved by the ethics committee, and all methods adhered to the applicable guidelines and regulations. Written informed consent was obtained from all patients undergoing the operation, and for patients under the age of 16, consent was obtained from a parent or legal guardian. Patients with an incomplete medical history and those who did not receive surgical treatment were excluded from the analysis. From June 2012 to December 2022, we obtained data on 85 clinically proven patients with surgery from our hospital’s electronic medical record system, and patients over 18 years of age (*n* = 58) were recontacted. An analysis of the surgical prognosis and pregnancy outcomes was done retrospectively over a lengthy period of time.The medical history was obtained, including demographic characteristics, gravity and parity, clinical presentation, physical examination, complications, MRI, US, and surgical findings. A descriptive analysis was performed with proportions, and an analysis of variance and standard deviation was done using t tests. Proportions were compared with the use of the χ² test or Fisher exact test, as appropriate. *P* < 0.05 was set for significance in all analyses.

### Imaging Assessment

Transvaginal (TVUS) or transrectal (TRUS) ultrasonography was used to assess each patient before surgery, and the examination was performed no later than two weeks earlier to the operations. Urinary tract ultrasonography was used to assess kidney abnormalities. Hematometra, hematocervix, hematocolpos, hematosalpinx, ovarian endometrioma, diagnosis of uterine abnormalities, and renal anomalies were among the ultrasound findings that were recorded.

A total of 63 patients underwent pelvic MRI examination. MRI descriptions and diagnoses of uterine anomalies, obstruction sites, possible communication between the obstructed and.contralateral side, endometriosis, any cystic structures, and other abnormal findings were also recorded

### Surgery

Surgical approaches were recorded for all patients undergoing gynecologic surgery. The surgical reports, the level of obstruction and communication between both sides, and other important findings were collected and compared to the imaging findings.

## Result

### Demographics and Clinical Manifestations

From June 2012 to December 2022, there were a total of 85 patients with HWWS enrolled in our study. We recommend categorising HWWS into complete or incomplete obstruction of the hemivagina depending on whether there is communication in the hemivagina or cervix. In our analysis, 27 patients were categorised as having complete obstruction, and 58 were categorised as having incomplete obstruction. The MRI found only 7/58 cases of site of communication, including 6 cases on the vaginal septum and 1 case on the cervix. There are distinct clinical differences between patients with complete obstruction of the hemivagina and those with incomplete obstruction [Table 1]. The majority of cases were presented after menarche, with the exception of a 12-year-old girl. This girl had a history of abdominal and pelvic discomfort for over a year prior to the onset of menstruation, and her symptoms had worsened progressively over time. HWWS occurred on the right side in 49 cases (57.6%) and on the left in 36 cases (42.4%).

The mean age at the onset of symptoms of complete obstruction was significantly younger than the mean age at the diagnosis of incomplete obstruction (*P* < 0.05). The mean age at diagnosis of complete obstruction was also significantly younger than the mean age at diagnosis of incomplete obstruction (*P* < 0.05). For complete obstruction, the median time between menarche and the onset of symptoms was 2.1 years, while for incomplete obstruction, it was 5.3 years. The incidences of intermittent mucopurulent discharge, irregular vaginal haemorrhage, and occasional examination findings indicative of complete obstruction were all significantly lower than those with incomplete obstruction(*P* < 0.05). Complete obstruction was significantly associated with dysmenorrhea and pelvic endometriosis compared with incomplete obstruction (*P* < 0.05). Furthermore, patients with complete obstruction are more likely to develop hematometros, hematosalpinx, and hemoperitoneum due to difficulty discharging menstrual blood (*P*=0.05).

**Table 1:** Clinical characteristics of patients with completely or incompletely obstructed hemivagina

clinical manifestations	complete obstruction	incomplete obstruction	*P*-value
Age at symptom onset (years)	14.2 ± 5.5	18.3 ± 6.1	*P* = 0.03
Age at time of diagnosis years)	16.2 ± 6.7	22.7 ± 7.7	*P* = 0.01
Duration between menarche and onset of symptoms (years)	2.1 ± 5.1	5.3 ± 4.9	*P* = 0.08
Dysmenorrhea (n/N (%))	19/27(70)	20/58(34)	*P* = 0.02
Intermittent mucopurulent discharge (n/N (%))	1/27(4)	19/58(33)	*P* = 0.03
Irregular vaginal hemorrhage (n/N (%))	2/27(7)	16/58(28)	*P* = 0.034
Prolonged menstrual duration^*^(n/N (%))	3/27(11)	10/58(17)	*P* = 0.465
Endometriosis (n/N (%))	9/27(33)	5/58(9)	*P* = 0.04
Pelvic inflammation (n/N (%))	3/27(11)	15/58(26)	*P* = 0.121
Asymptomatic (n/N (%))	1/27(4)	12/58(21)	*P* = 0.043
Hematometra, hematosalpinx and hemoperitoneum	14/27(52)	16/58(28)	*P* = 0.05

* menstrual duration ≥ 8 days

### Uterine Anomalies, Obstruction Site and Communications

**Fig. 1:**
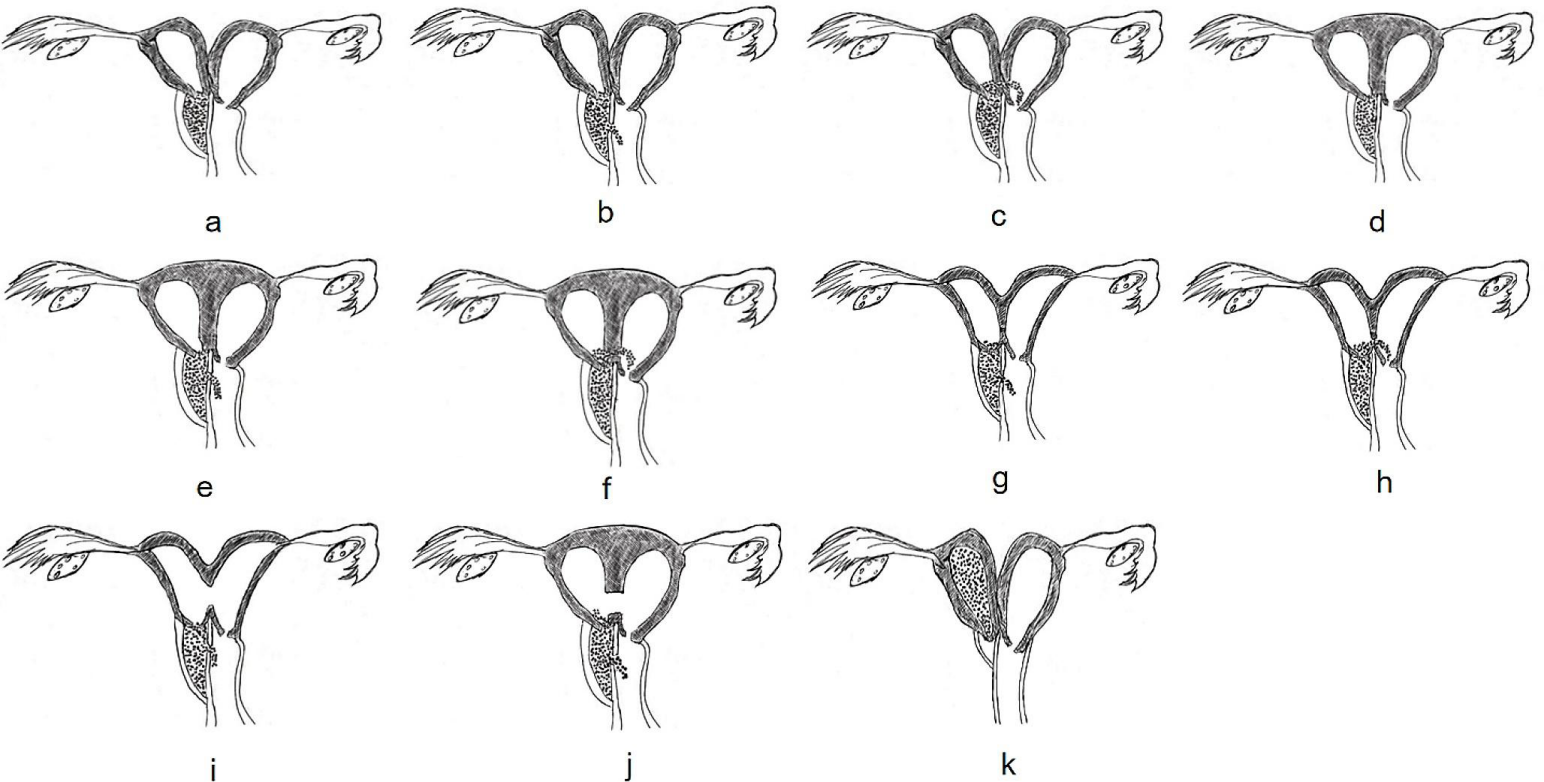
(**a**) uterus didelphys and obstructed hemivagina; (**b**) uterus didelphys and obstructed hemivagina with microperforation; (**c**) uterus didelphys and obstructed hemivagina with a communicating cervix; (**d**) obstructed hemivagina and complete septate uterus; (**e**) obstructed hemivagina with microperforation and complete septate uterus; (**f**) obstructed hemivagina and complete septate uterus with a communicating cervix; (**g**) uterus bicornuate bicollis and obstructed hemivagina with microperforation; (**h**) uterus bicornuate bicollis with a communicating cervix and obstructed hemivagina; (**i**) obstructed hemivagina and bicornuate bicollis uterus with open cavity; (**j**) incomplete septate uterus and obstructed hemivagina with microperforation; (**k**) obstructed hemivagina, hemiuterus, and single cervix with separate contralateral patent hemiuterus, cervix, and vagina

**Fig. 2:**
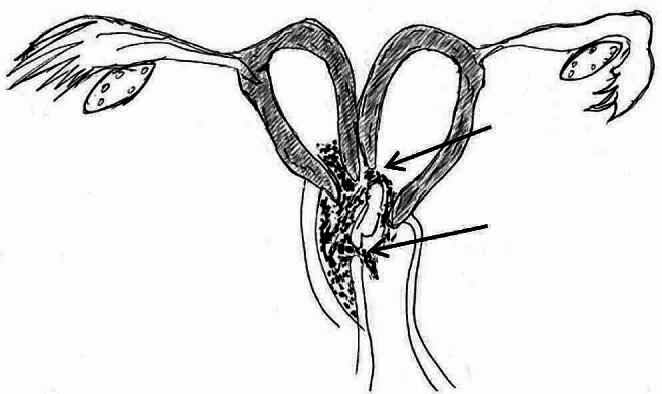
Uterus didelphys and blind hemivagina with connecting holes in the vaginal septum and between the two cervices (the black arrow indicating the location of the communications)

**Fig. 3:**
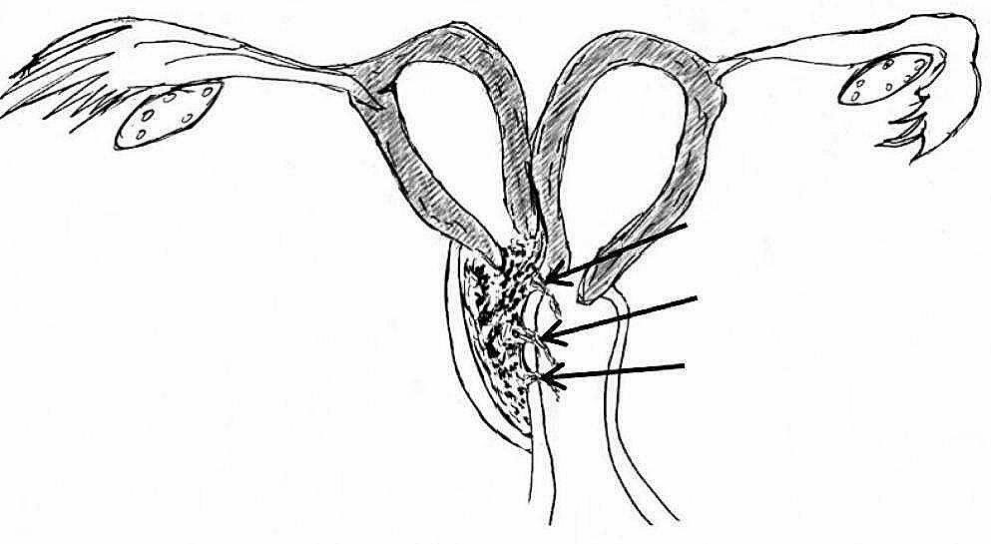
Uterus didelphys and blind hemivagina with three communicating holes in the vaginal septum (the black arrow indicating the location of the communications)

There were various combinations of uterine anatomy, obstruction site, and communication. 56/85 patients (65.9%) had uterus didelphys and obstructed hemivagina (22/56 cases with microperforation in vaginal septum and 13/56 cases with communication in cervix ); 23/85 patients (27.1%) had complete septate uterus duplicated cervices and obstructed hemivagina (13/23 case with communicant hemivagina and 4/23 with communication in cervix); 4/85 patients (4.7%) had a uterus bicornuate bicollis and obstructed hemivagina (2/4 cases with communicant hemivagina and 2/4 with communication in cervix); 1/85 patients (1.2%) had obstructed hemivagina with microperforation and bicornuate bicollis with open cavity; 1/85 patients (1.2%) had incomplete septate uterus duplicated cervices and obstructed hemivagina with microperforation. The various combinations of uterine anomalies, obstruction sites, and communications are shown in Fig. 1. All patients had bilaterally located ovaries that showed all stages of follicular development on their surface. There were connecting holes in the vaginal septum and between the two cervices in 2 patients (Fig. 2), one with not only progressive dysmenorrhea but also adenomyosis and the other with sporadic clear, odourless vaginal fluid. One patient came to our hospital because of menopause following an abortion, and she had three communicating holes in the vaginal septum (Fig. 3). They did not have the typical hemoglobinopathy on imaging findings.

### Urologic and Ureteral Anomalies

The results in terms of urological anomalies are summarized in Table 2. 81 patients had ipsilateral kidney agenesis, 2 had an ipsilateral dysplastic kidney, and 2 patients have the bilateral presence of normal kidneys. We found ectopic ureter insertion into the ipsilateral obstructed hemivagina in 4 cases, confirmed with ureteroscopy. There were 2 cases of simple ipsilateral paravaginal cysts, which were shown on MRI as a cystic structure lateral to the vagina and cervix and posterior to the bladder, neither inserted into the obstructed cervix nor the obstructed vagina, and of which 1 case was confirmed by surgical pathologic evaluations. In one case, ultrasound revealed a cystic structure in the bladder wall formed by distal ureteral remnants. Table 3 presents the different combinations of uterine anomalies, unilateral cervico-vaginal obstruction, and renal anomalies.

**Table 2:** Urologic anomalies

Anomaly	*n*
Absence of the ipsilateral kidney	81
Permeable ectopic ureter ending	2
Blind ectopic ureter	2
Cystic structure in the bladder wall formed by ipsilateral ureter	1
Ipsilateral dysplastic kidney	2
Permeable ectopic ureter ending	2
Bilateral presence of normal kidneys	2

**Table 3:** Number of different combination of uterine anomalies, unilateral cervico-vaginal obstruction, and renal anomalies

Obstruction site	Total	Uterine anomalies					Laterality		Renal anomalies		
		Didelphys	Septate	Bicornuate bicollis	Bicornuate bicollis with open cavity	Incomplete septate	Right	Left	Agenesis	Dysplasia	Normality
Vaginal septum	81	53	23	3	1	1	46	35	77	2	2
Cervical obstruction	4	4	0	0	0	0	3	1	4	0	0
Total	85	57	23	3	1	1	49	36	81	2	2

### Other Findings

**Fig. 4:**
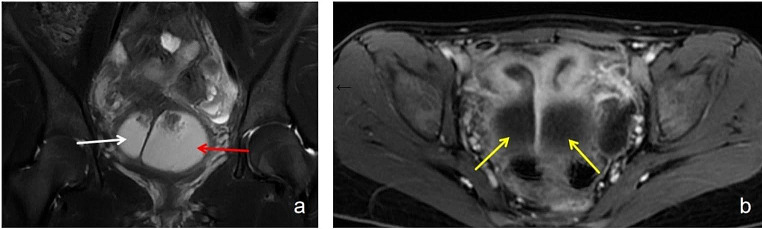
MRI of the pelvis. (**a**). Bilateral hematocolpos (white straight arrow suggesting hematocolpos caused by the oblique septum and red straight arrow indicating hematocolpos caused by imperforate hymen. (**b**). Bicornuate bicollis uterus (straight arrow suggesting bilateral hematometra). The pelvic MRI showed bicornuate bicollis uterus, with bilateral hematometra, and bilateral hemivagina (commuicating with the hemiuteru), distended by a content whose signal was methemoglobin

**Fig. 5:**
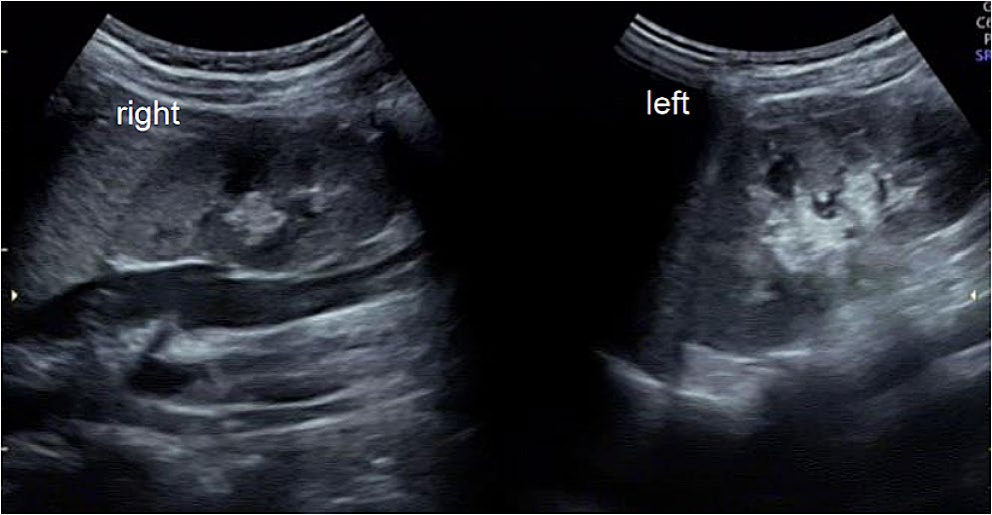
Renal ultrasound suggested bilateral normal kidneys

An 11-year-old girl was admitted to the hospital due to lower abdominal pain and the presence of a palpable abdominal mass. Transanal three-dimensional ultrasound and pelvic MRI revealed uterus didelphys and double vaginal effusion (Fig. 4). A renal ultrasound revealed that both kidneys were of a normal size (Fig. 5). Under the diagnostic suspicion of hymen atresia and partial vaginal atresia, the patient was referred to the hospital for further treatment. A colpo-hysteroscopy was then performed to confirm the clinical hypothesis. After a vaginoscopy revealed hymen atresia, the hymen was cut with curved forceps, and there was a large amount of blood coming out. And hysteroscopic examination suggested a left-sided patent hemiuterus with a single uterine cervix, uterine tube, ostium, and right lateral vaginal wall bulging with a small communication with normal hemivagina. A hysteroscopic incision of the bulging oblique vaginal septum was performed medially from the most prominent point of the septum up to the left cervix and then down to the low edge of the oblique septum. The previously obstructed hemiuterus was hysteroscopically examined, confirming the presence of a cervix and a tubular cavity with one tubal ostium. Finally, the patient was diagnosed with HWWS and hymen atresia.

## Surgery

All patients underwent resection of the vaginal septum, and laparoscopic diagnosis and treatment were performed on 15 patients at the same time.

### Fertility

During the first visit, 38 successful pregnancies were reported by 19 patients; 8 of the patients had term deliveries, and they had experienced 24 miscarriages. Pregnancy occurred in the uterus ipsilateral to the hemivaginal septum in 6 (16%) cases and in the uterus contralateral to the hemivaginal septum in 32 (84%) cases. Among the 58 patients above 18 years of age contacted by telephone, only 38 answered. There was no recurrence of obstruction, and, regarding fertility, 15 successful pregnancies were reported by 11 patients, with 6 within the former obstructed hemiuterus. The remaining patients had not been assigned a child project yet.

## Discussion

Agenesis of the kidneys, hemivaginal obstruction, and uterus didelphys are the three features of the uncommon congenital condition known as HWWS [10, 11]. It is most commonly diagnosed in puberty due to dysmenorrhea, vaginal discharge, pelvic or abdominal pain, but more rarely, it can present in neonates or adults with primary infertility, pyometra, urinary obstruction, and ischiorectal swelling [8, 12]. Because of the low incidence of HWWS and the lack of specificity of clinical symptoms, it is easy to miss diagnose and misdiagnose, resulting in endometriosis, pelvic adhesion, infertility, and other complications [8].

Communication may exist between the obstructed and contralateral sides. In this study, communicant HWW syndrome was more common (68%) than noncommunicant HWW syndrome (32%). An ultrasound or an MRI is not very sensitive to detecting communications in the vagina, especially small holes. One study showed that MRI could identify only 20% of communications [13]. In fact, the presence, location, and quantity of communication and ectopic ureter insertion into the ipsilateral obstructed hemivagina are mainly determined by careful examination with hysteroscopy. In our study, there were 27 patients with no communication between the uterus and vagina, 38 patients with communication in the vaginal septum, 18 patients with communication in the cervix, and 2 patients with communication both between the vaginal septum and cervix. And one patient had three small holes in the vaginal septum. Earlier literature [14, 15] reported HWWS to be more common on the right than on the left, with a ratio of 2:1 [16], whereas our study found it to be 1.4:1.

Several non-specific manifestations of HWWS occur after menarche or around that time. Whether the vaginal oblique septum was completely or partially obstructed affected the clinical presentation. There was a younger onset age for noncommunicant HWW syndrome (14.2 years) than for communication (18.3 years), which was in accordance with the literature [17, 18]. Complete obstruction is characterized by dysmenorrhea shortly after menarche and hematomatre caused by the hemivagina obstruction, while incomplete obstruction is characterized by abnormal vaginal discharge, such as blood or pus. Asymptomatic conditions are more common in patients without obstruction, which may be explained by the menstrual blood discharged through communication.

In Sampson’s opinion, retrograde menstruation and implantation account for most cases of endometriosis. An earlier study found that 19% of HWWS patients had pelvic endometriosis [19] and that the rate of endometriosis was higher among patients with complete obstructions than among those with incomplete obstructions, suggesting that the obstructed hemivaginal septum was the direct cause of endometriosis. Endometriosis can be diagnosed early, especially in adolescents, to prevent further disruption of the normal anatomy and fertility loss. Endometriosis is associated with an obstructive genital anomaly found in adolescents, for which the severity depends on the delay in establishing the diagnosis. These lesions may regress spontaneously, sometimes completely, when the anomaly has been surgically treated [20].

There are some uterine malformation variants of HWWS, including the didelphys uterus, septate uterus, bicornuate uterus, and bicornuate and complete septate uterus. Most cases of HWWS were of the didelphys uterus, a classic variety of uterine deformity, according to L. Fedele et al. [9]. Our findings support and expand on earlier research. There are different surgical options for different uterine malformation variants, which may be related to conceiving or maintaining a pregnancy in the future. A didelphys uterus has not been associated with infertility or pregnancy complications, and surgical intervention is not routinely recommended [21]. In contrast, for a septate uterus, pregnancy outcomes are significantly improved after uterine septum resection [22].

Anomalies of the kidney are connected to ipsilateral obstructive Müllerian abnormalities in around 50% of cases [23]. The right side is where kidney abnormalities are more common [24, 25], which is consistent with our findings. Kidney abnormalities include renal agenesis, renal dysplasia, renal hypoplasia, duplicated kidneys, and multicystic dysplastic kidneys, according to earlier reports [25–27]. In our study, we discovered ipsilateral renal agenesis, ipsilateral dysplastic kidneys, and the bilateral presence of normal kidneys. Regarding the urinary system, in some HWWS cases, attention should be given not only to the renal abnormalities but also to the existence of ureteral remains. Cystoscopy is required when imaging points to the existence of ureteral remains. A case of HWWS with left renal dysplasia and a left ectopic ureter connecting with the vagina was described [28]. Adi et al. [29] described a case of HWWS with a urethrovaginal fistula. We found ectopic vaginal ureteral ending in 4 cases. It could be argued that the primary movements in the embryogenesis of this malformation is a unilateral anomaly of incorporation of the distal part of the wolffian duct within the presumptive bladder. Because of close relations between wolffian and müllerian ducts in this region, this may lead to anomalies of the unilateral müllerian duct, leading to anomalies in fusion processes of the both müllerian ducts [26, 30, 31]. Moreover, the wolffian anomaly of incorporation may also induces an anomaly in the incorporation of the ureteric bud within the presumptive bladder [30]. There is no doubt that the presentations of the urogenital malformations in HWWS are diverse.

Ultrasound has traditionally been the first imaging modality chosen for the diagnosis of HWWS because it is affordable, doesn’t expose users to radiation, and provides better images of the uterus and adnexa [32]. In recent years, 3D ultrasound has become the method of choice for the diagnosis and assessment of congenital uterine anomalies [33]. MRI is currently regarded by several writers as the gold standard for HWWS diagnosis and surgical planning [34]. The goal of surgery is to remove the oblique vaginal septum, observe whether there is a fistula, and rule out any further problems. Total excision of the vaginal septum is the most important treatment for HWWS. MRI has excellent soft tissue resolution and a broad imaging field of view and is able to evaluate uterine malformations, hematocolpos, hematometra, hematosalpinx, and ovarian or pelvic endometriosis by imaging in different orientations, dimensions, and parameters, which is of great significance for early clinical diagnosis and treatment [35, 36]. Since MRI is the best imaging modality for endometriosis, the relationship between HWWS and endometriosis also favours this condition [35]. It is important to note that CT is not the first option for female pelvic examination, but it may still be utilised as an emergency examination for patients with severe lower abdomen discomfort if the ultrasound diagnosis is unclear and the MRI examination takes a long time [32].

When adolescent girls come to the emergency room with an acute abdomen, appendicitis, hymen atresia, and adnexal torsion are indeed the main causes of predisposition and diagnosis [30]. But the presence of HWWS should be considered when renal agenesis is found during systematic abdominal and gynecologic examinations completed by radiologic evaluation. In addition, we should take into account the occurrence of HWWS merging these situations. Patients who have both HWWS and hymen atresia frequently overlook HWWS because the acute obstructive symptoms brought on by hymen atresia conceal those brought on by the oblique vaginal septum, and the obstructive symptoms are much diminished following the release of hymen atresia.

Clinicians are not fully aware of HWWS since it is rare and has unusual clinical presentations. Some patients with HWWS delay seeking medical attention, and HWWS is discovered only when infertility occurs. Such patients are vulnerable to misdiagnosis and missed diagnosis because they ignore their own clinical signs. Even after menstruation, HWWS is frequently misdiagnosed. Several reasons may justify the delayed symptoms: first, the vagina can accommodate a large volume of blood due to its distention properties; second, some of the blood is absorbed between menses [32]; third, oral contraceptives and anti-inflammatory drugs are generally recommended to relieve dysmenorrhea [37, 38]; and finally, some patients who present with isolated vaginal discharge are mistakenly treated with long-term antibiotics.

Herlyn-Werner-Wunderlich syndrome is an exceedingly uncommon conglomeration of congenital anomalies. Timely and efficacious surgical intervention will undeniably enhance quality of life, avert complications, and maintain long-term fertility. A comprehensive preoperative evaluation of obstruction is pivotal in determining the most advantageous surgical methodology. A robust association is evident between the existence of complete obstruction and symptoms, age at symptom onset, and diagnosis. Additionally, careful consideration should be given to the height of communication in the hemivagina, with symptoms manifesting at an earlier stage when the obstruction is higher, as menstrual blood drainage from a high communication is not as smooth as from a low one. Further work is needed to determine whether the level of communication will affect the onset and seriousness of clinical manifestations.

### Limitation

There were some limitations in our study. Our study was a single-center retrospective study in which a relatively small number of patients were included. In addition, Some cases were diagnosed and treated many years ago. Therefore, a surgical approach could vary not only due to anatomical differences, but also due to medical advances and the possibility of delayed diagnosis. Further studies are required to avoid selection and observational bias, and long-term prospective or randomized control trials are necessary to provide optimal clinical evidence to better manage this disorder.

## Electronic Supplementary Material

Below is the link to the electronic supplementary material.

Supplementary Material 1

Supplementary Material 2

## Data Availability

All supplemental materials for this article is available from the corresponding authors based on reasonable request.
