# Supplementary figures and images for: Clinical and Imaging Characteristics of Herlyn-Werner-Wunderlich Syndrome: a Comprehensive Analysis
Source: Reprod Sci. 2024 Jun 21;31(11):3343–50. doi: 10.1007/s43032-024-01628-2 (PMC11527966; doi:10.1007/s43032-024-01628-2)

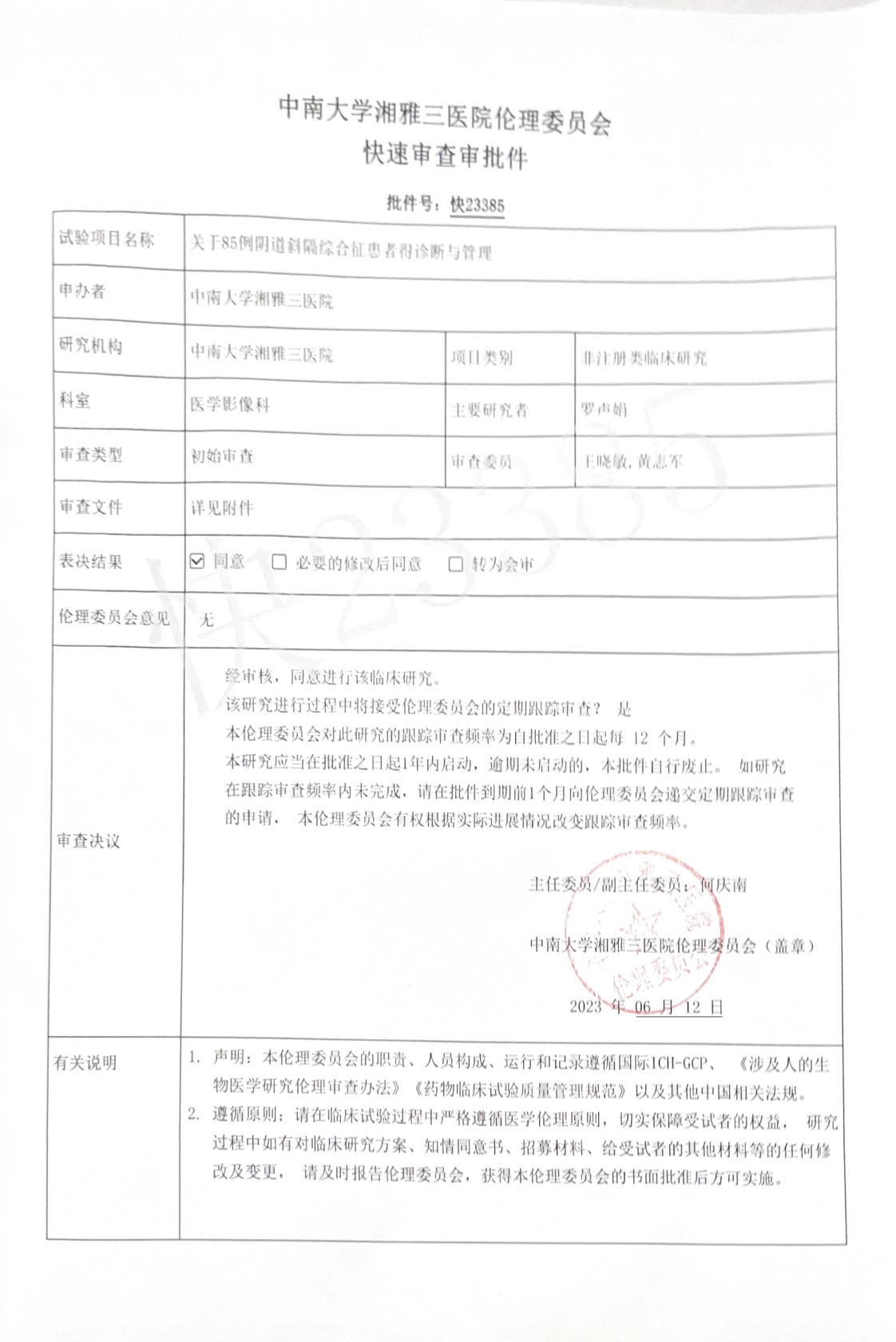

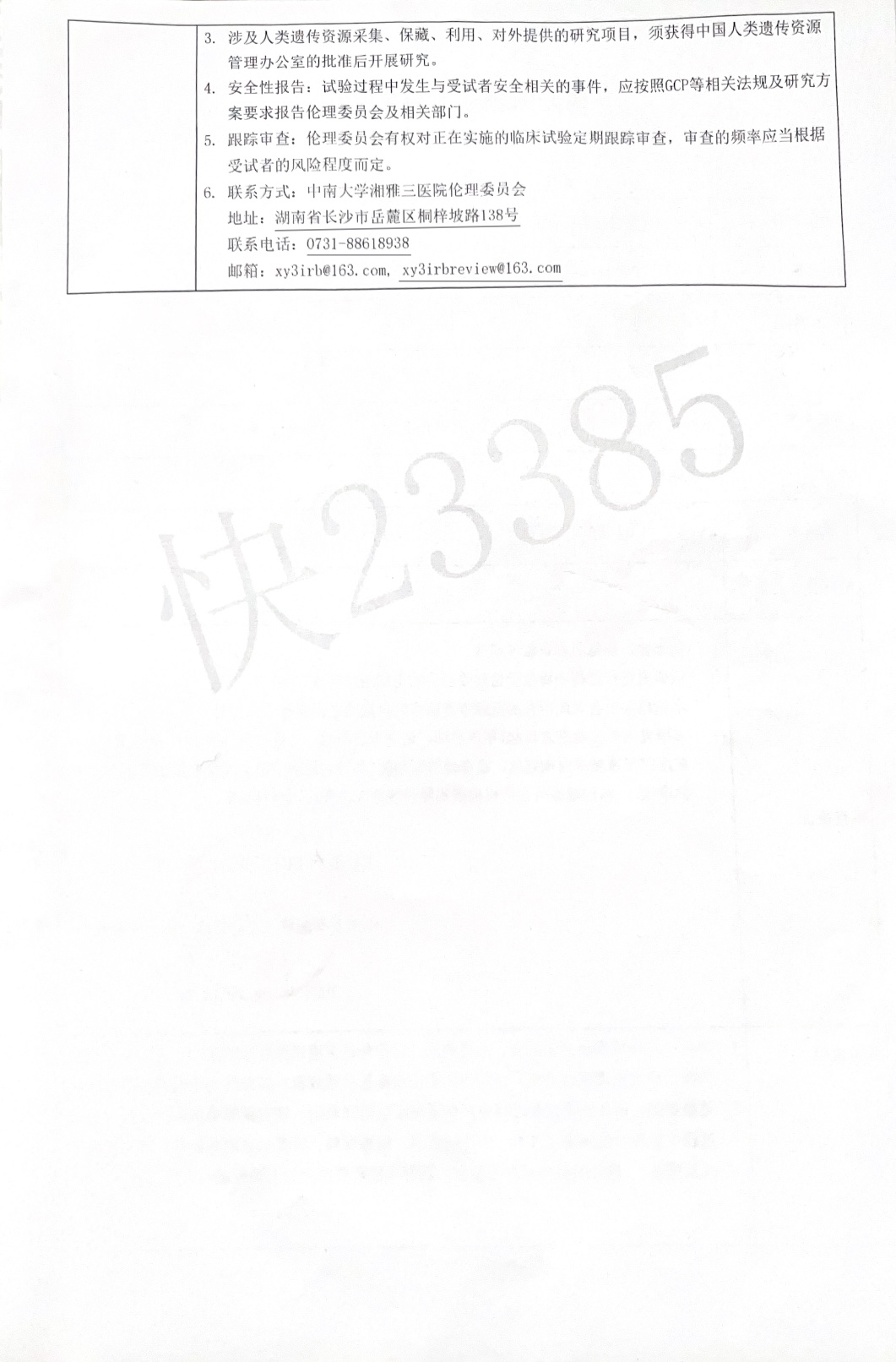

Supplement: Supplementary file 1 — Supplementary Material 1 [file 43032_2024_1628_MOESM1_ESM.docx]
