# Supplementary material for: Clinical and Imaging Characteristics of Herlyn-Werner-Wunderlich Syndrome: a Comprehensive Analysis
Source: Reprod Sci. 2024 Jun 21;31(11):3343–50. doi: 10.1007/s43032-024-01628-2 (PMC11527966; doi:10.1007/s43032-024-01628-2)
Supplement: Supplementary file 2 — Supplementary Material 2 [file 43032_2024_1628_MOESM2_ESM.docx]

Ethics Committee of the Third Xiangya Hospital of Central South University

Fast Review and Approval Documents

Document number: Quick 23385

| Experimental Project Title | Clinical and Imaging Characteristics of Herlyn-Werner-Wunderlich Syndrome: A Comprehensive Analysis | | |
| --- | --- | --- | --- |
| Applicant | The Third Xiangya Hospital of Central South University | | |
| Research institution | The Third Xiangya Hospital of Central South University | Project category | Non-registered clinical research |
| Department | Department of Imaging | Primary researcher | Shengjuan Luo |
| Review Type | Initial review | Review committee | Xiaomin Wang, Zhijun Huang |
| Review Document | 1. Signature Page of the Proposal. 2. Research plan. 3. Informed Consent Form. 4. Researcher Resume. 5. Researcher Conflict of Interest Statement. 6. Blank form. 7. List of Registrants. | | |
| Result | Agree | | |
| Ethics Committee Opinion | Nothing | | |
| Review of Resolutions | After review, it has been approved to conduct this clinical research.  Will the study undergo regular monitoring and review by the ethics committee during the process? Yes.  The frequency of follow-up review of this study by this ethics committee is every 12 months from the date of approval.  This research shall be started within 1 year from the date of approval, and if it is not started within the time limit, this approval document shall be abolished by itself. If the research is not completed within the frequency of follow-up review, please submit an application for regular follow-up review to the Ethics Committee one month before the expiration of the approval document, and the Ethics Committee has the right to change the frequency of follow-up review according to the actual progress.  Chairman/Vice Chairman: Qinnan He  12 June 2023 | | |
| Instructions | 1. Statement: The responsibilities, composition, operation and records of this ethics committee comply with the international ICH-GCP, the Measures for the Ethical Review of Biomedical Ethics Involving Humans, the Good Practice for Drug Clinical Trials and other relevant Chinese regulations. 2. Follow the principles: Please strictly follow the principles of medical ethics in the process of clinical trials, effectively protect the rights and interests of subjects, and if there are any modifications and changes to the clinical research protocol, informed consent form, recruitment materials, other materials for subjects during the research process, please report to the ethics committee in a timely manner, and obtain the written approval of the ethics committee before implementation. 3. Research projects involving the collection, preservation, utilization, and external provision of human genetic resources must be carried out with the approval of the Chinese Human Genetic Resources Management Office. 4. Safety report: If an incident related to the safety of subjects occurs during the trial, it should be reported to the ethics committee and relevant departments in accordance with GCP and other relevant regulations and research protocol requirements. 5. Follow-up review: The ethics committee has the right to conduct regular follow-up reviews of ongoing clinical trials, and the frequency of review should be determined according to the risk level of the subject. 6. Contact information: Ethics Committee of the Third Xiangya Hospital of Central South University   Address: No. 138, Tongzipo Road, Yuelu District, Changsha City, Hunan Province  Contact number: 0739-88618938  Email: [xy3irb@163.com,](mailto:xy3irb@163.com,) [xy3irbreview@163.com](mailto:xy3irbreview@163.com) | | |
